# Association Analysis of* MET* Gene Polymorphism with Papillary Thyroid Carcinoma in a Chinese Population

**DOI:** 10.1155/2015/405217

**Published:** 2015-11-15

**Authors:** Lifeng Ning, Yaqin Yu, Xiaoli Liu, Lizhe Ai, Xin Zhang, Wenwang Rao, Jieping Shi, Hui Sun, Qiong Yu

**Affiliations:** ^1^Department of Epidemiology and Biostatistics, School of Public Health, Jilin University, Changchun 130021, China; ^2^National Research Institute for Family Planning, Beijing 100081, China; ^3^Jilin Provincial Key Laboratory of Surgical Translational Medicine, Department of Thyroid and Parathyroid Surgery, China-Japan Union Hospital, Jilin University, Changchun 130033, China

## Abstract

To investigate the association of* MET* SNPs with gender disparity in thyroid tumors, as well as the metastasis and prognosis of patients, 858 patients with papillary thyroid carcinoma (PTC), 556 patients with nodular goiter, and 896 population-based normal controls were recruited. The genotyping of* MET* SNPs was carried out using the Sequenom MassARRAY system. The distribution of* MET* SNPs (rs1621 and rs6566) was different among groups. Gender stratification analysis revealed a significant association between the rs1621 genotype and PTC in female patients (*P* = 0.037), but not in male patients (*P* > 0.05). For female patients, the rs1621 AG genotype was significantly higher in patients with PTC than in normal controls (*P* = 0.01) and revealed an increasing risk of PTC (OR: 1.465, 95% CI: 1.118–1.92). However, association analysis of the rs1621 genotype with metastasis and prognosis revealed no significant correlation in both male and female patients. The findings of our study showed that polymorphism of SNP locus rs1621 in* MET* gene may be associated with gender disparity in PTC. Higher AG genotypes in rs1621 were correlated with PTC in female patients, but not in male patients.

## 1. Introduction

Papillary thyroid carcinoma (PTC) is the most common form of thyroid malignancy, which generally has a good prognosis and accounts for approximately 80–85% of all thyroid carcinomas [1–3]. However, this type of cancer may cause distant metastasis and be more aggressive in older patients [4]. Established risk factors for PTC include ionizing radiation, positive family history, and thyroid nodular disease [5]; but these factors do not appear to account for the increasing incidence of PTC [6]. Studies have shown that age, gender, Hashimoto's thyroiditis, thyroid-stimulating hormone concentrations, solitary nodularity, and anti-thyroglobulin antibodies positivity are known risk factors for PTC development [7, 8]. Other studies have proposed that genetic factors may also contribute to the risk of PTC [9, 10].

Gender difference in incidence, aggressiveness, and prognosis has been well-established in thyroid cancer. The incidence of thyroid cancer has been reported to be three to five times more frequent in women, and this gender difference is particularly obvious for women of reproductive age [11]. Gender disparity in thyroid cancer has also been known to be specific to the histologic subtype of thyroid cancer, with the more commonly differentiated thyroid cancer of follicular cell origin including PTC in women. However, the potential reason for this disparity is poorly understood. Genetic analysis such as single-nucleotide polymorphism analysis has been suggested to be helpful in better understanding the molecular basis for gender disparity in thyroid and other cancers [12].

The cellular mesenchymal-epithelial transition (*MET*) factor is a plasma membrane tyrosine kinase receptor that has low activity in normal tissues but is dysregulated in many tumor types [13]. It can be activated in tumor cells through mutation, amplification, and overexpression [14]. The dysregulated activation of* MET* kinase may correlate with the aggressiveness of tumor growth and metastasis [15]. The role of* MET* mutation in human cancer was first established in papillary renal carcinoma [16]. Mutations of the* MET* protooncogene have also been described in several other types of human cancers and were suggested to correlate with tumor metastasis [17–19]. Therefore, the present study was designed to investigate the association of* MET* single-nucleotide polymorphisms (SNPs) with the gender disparity of thyroid tumors, as well as the metastasis and prognosis of PTC in the Chinese population.

## 2. Material and Methods

### 2.1. Subjects

The study subjects comprised 858 patients with PTC (43.7 ± 9.17, 208 males/650 females), 556 patients with nodular goiter (NG, 48.6 ± 9.94, 131 males/425 females), and 896 population-based normal controls (NC, 43.7 ± 9.06, 219 males/677 females). All patients were recruited from the China-Japan Union Hospital of Jilin University during August 2012 to December 2014. Healthy individuals were collected from The First Hospital of Jilin University during the same period. Patients with PTC and NG were diagnosed according to the revised American Thyroid Association management guidelines for patients with thyroid nodules and differentiated thyroid cancers [20]. Age- and gender-matched control subjects were from the general population, who were free from thyroid diseases, diabetes, and other endocrine system diseases. All patients and control subjects came from the Han population of Northern China. This study was reviewed and approved by the Medical Ethics Committee of the School of Public Health, Jilin University; and written informed consent was obtained from all participants.

### 2.2. DNA Extraction and SNP Genotyping

SNP data located in the* MET* gene were downloaded from the HapMap database. Two tag SNPs of the gene, rs1621 and rs6566, were obtained through Haploview 4.2 (population: CHB, *R* square cutoff: 0.8, MAF cutoff: 0.1, and *D*′ = 1). Blood samples were collected from all participants, and the whole DNA genome was isolated using a DNA extraction kit (Beijing Kangwei Century Biotech Co., Ltd., China) according to standard protocols. The concentration and purity of the DNA samples were determined by a UV-260 spectrophotometer (Shimadzu, Kyoto, Japan). SNP genotyping was carried out using the Sequenom MassARRAY platform (San Diego, CA, USA). Primer sequences used were as follows: rs1621-F: ACGTTGGATGACCCTGAGCAGAACTTTGTG, rs1621-R: ACGTTGGATGGTAACCTACACCACATGCAC, and rs6566-F: ACGTTGGATGCTGGCAATAACCACTATCAG; rs6566-R: ACGTTGGATGACTGAATGGTACTTCGTATG. Polymerase chain reaction (PCR) analysis was performed with Taq DNA polymerase (Tiangen Biotech Co., Ltd., Beijing, China). PCR condition was 94°C for 15 minutes to perform a hot-start, followed by denaturing at 94°C for 20 seconds, annealing at 56°C for 30 seconds, extension at 72°C for one minute for 45 cycles, and incubation at 72°C for three minutes. Then, shrimp alkaline phosphatase (SAP) reaction was performed by incubating the PCR product with SAP (Sequenom, Inc., San Diego, CA, USA) at 37°C for 40 minutes, followed by inactivation at 85°C for five minutes. The iPLEX extension reaction was performed at 94°C for 30 seconds and for five seconds, followed by 40 cycles at 52°C for five seconds and five cycles at 80°C for five seconds and at 72°C for three minutes. The product was desalted by the addition of resin in a 384-dimple plate, mixed, resuspended, and centrifuged to separate the extension products from the resin. The completed products were analyzed using the MassARRAY Typer software version 4.0 (Sequenom, USA).

### 2.3. Statistical Analysis

All statistical analyses were conducted using the “genetics” and “dgc.genetics” packages running in the R software environment (version 3.0.2). Hardy-Weinberg equilibrium was examined in control samples by Pearson's *χ* ^2^-test. The interaction between the* MET* gene and gender was examined by logistic regression using an additive model. All statistical tests were two-sided. A *P* value < 0.05 was considered statistically significant.

## 3. Results

There was no apparent difference with respect to gender among the three groups (*P* = 0.991). The age of patients in the NG group was comparatively older than the age of patients in the PTC and NC groups (*P* < 0.001), while there was no significant difference between the PTC and NC groups (*P* = 0.947). The SNP distribution satisfied the Hardy-Weinberg equilibrium (*P* > 0.05). The allele and genotype frequencies of* MET* in the PTC, NG, and NC groups are listed in Table 1.* MET* SNPs (rs1621 and rs6566) were differentially distributed among the groups.

Interactions between* MET* SNPs (rs1621 and rs6566) and PTC were analyzed by stratifying the patients according to gender. The* MET* SNP rs1621 genotype was significantly associated with PTC in female subjects (*P* = 0.037), but not in their male counterparts (*P* > 0.05), as shown in Table 2. There was no significant association between rs6566 and PTC in both male and female genders (*P* > 0.05). Therefore, the genotype frequency of rs1621 was analyzed in the female and male part of the PTC, NG, and NC groups, respectively. Multiple comparison adjustments were made using Bonferroni correction (*α* = 0.017 = 0.05/3). Among female patients, the rs1621 AG genotype was significantly higher in patients with PTC when compared with normal controls, while the frequency of genotypes AA and GG was comparatively lower (*P* = 0.01). There was no significant difference in rs1621 genotype distribution between the NG and NC groups or between the PTC and NG groups (*P* = 0.908 and *P* = 0.078, resp.). By contrast, no significant difference was found among male patients.

Differences in rs1621 genotype frequencies in female patients in each group were assessed by an additive model of logistic regression using the major allele as a reference. AG genotype revealed an increasing risk of PTC in female patients (OR: 1.465, 95% CI: 1.118–1.92), as shown in Table 3, indicating an interaction between gender and* MET* SNP (rs1621) in PTC patients. Further analysis of the association between the genotype frequencies of* MET* SNP (rs1621) and PTC metastasis or prognosis of patients did not reveal a significant correlation in both male and female patients (Table 4).

## 4. Discussion

*MET* polymorphisms are associated with cancer risk [21, 22]. The presence of adenosine (A) at SNP rs1621 has been reported to increase the risk of cancer development [23]. SNP rs1621 in the seed-matching sequence of* MET* has been suggested to affect the activity of miR-199a, which mediates the downregulation of the* MET* gene through targeting the 3′-UTR [24]. Furthermore, SNP rs1621 was also selected to investigate its effect on breast cancer risk [25]. SNP rs6566, located in* MET*, has also been investigated for gastric cancer risk [26]. In this study, these two tag SNPs (rs1621 and rs6566) were obtained by Haploview 4.2 (population: CHB, *R* square cutoff: 0.8, MAF cutoff: 0.1, and *D*′ = 1). Our study revealed a significant gender difference in the genotype frequencies of* MET* SNP rs1621 among patients with PTC and NG and NC. Multiple comparisons between groups further confirmed the statistical difference in rs1621 genotype frequency for female patients in the PTC and NC groups. Genotype AG in rs1621 increased the risk of PTC in female patients, while there was no significant difference between groups for male patients. A higher AG genotype frequency was the significant risk factor for PTC in female patients, compared with male patients. However, no significant association was found between rs1621 genotype frequency and the metastasis and prognosis of PTC in both male and female patients.

Disparity between genders in incidence, disease aggressiveness, therapy responsiveness, and prognosis has long been observed in a variety of gender-nonspecific cancers [27]. As the most common cancer of the endocrine system, thyroid cancer has a well-established gender disparity in incidence, aggressiveness, and prognosis. It has been reported to be the seventh most common malignancy in women, but not among the most common 15 cancers in men [28]. Several hypotheses have been proposed for gender differences in thyroid cancer initiation and progression. Reproductive, menstrual, and environmental factors have been hypothesized to account for this gender-specific disparity [12]. However, other studies have also indicated that dietary, environmental, and reproductive factors, as well as frequent activating somatic mutations, do not appear to contribute to this difference in PTC, the most common type of thyroid cancer [29]. Female gender hormones have been suggested to play a crucial role in the carcinogenesis of cancers [30], while hormonal exposure did not affect the innate characteristics of the tumor [31]. Gender disparity in cancers has been postulated to be due to yet unidentified molecular factors. The identification of these factors may help us better understand the biological behavior of cancers, which is essential in the development of effective strategies for cancer diagnosis and treatment.

Characterized by early metastasis and multifocal involvement, PTC exhibits a highly invasive behavior [32]. The* MET* protooncogene encodes a tyrosine kinase receptor that is known to influence cell invasion. It has been demonstrated to be significantly overexpressed in PTC [33] and has been suggested to play an important role in PTC invasion and metastasis [34].* MET* mutations have been reported in various types of cancers and were found to be correlated with tumor metastasis [17–19, 35]. However, other studies also revealed low mutation frequencies [36] or the mutation was not correlated with the progression of the disease [37]. In thyroid cancer,* MET* alteration has been reported to be relatively frequent in differentiated types [38].* MET* SNPs rs1621 and rs6566 were reported in our study, and stratification analysis according to gender revealed an increased risk of PTC in female patients with the AG genotype in rs1621, which has been previously reported to correlate with chronic rhinosinusitis [39]. By contrast, there was no significant association in male patients.* MET* single-nucleotide variants have been identified in PTC with or without distant metastases and were suggested to correlate with the aggressive behavior of the disease [40]. Therefore, the association between rs1621 and tumor metastasis, as well as the prognosis of patients, was analyzed in PTC patients. Our results revealed that there was no obvious correlation between rs1621 and the metastasis and prognosis of PTC patients in either the male or female gender. Further studies are needed to uncover the gender disparity of* MET* SNP rs1621 in PTC patients.

In conclusion, our study revealed that the polymorphism of SNP locus rs1621 in the* MET* gene may be associated with gender disparity in PTC. Higher AG genotypes in rs1621 were correlated with PTC in female patients, but not in their male counterparts. However, SNP rs1621 was not correlated with metastasis and prognosis in both male and female PTC patients. Further studies are needed to better clarify the association between* MET* polymorphism and gender disparity in PTC and its potential mechanisms.

## Figures and Tables

**Table 1:** Allele and genotype frequency of *MET* SNPs in patients with PTC and NG and NC.

*MET*	PTC (*n* = 858)	NG (*n* = 556)	NC (*n* = 896)	*p* value
rs1621				
AA	657 (76.6%)	449 (80.8%)	724 (80.8%)	0.050
AG	193 (22.5%)	98 (17.6%)	158 (17.6%)	
GG	8 (0.9%)	9 (1.6%)	14 (1.6%)	
A	1507 (87.8%)	996 (89.6%)	1606 (89.6%)	0.176
G	209 (12.2%)	116 (10.4%)	186 (10.4%)	
rs6566				
AA	178 (20.7%)	131 (23.7%)	214 (24.1%)	0.161
AG	433 (50.3%)	273 (49.4%)	458 (51.6%)	
GG	250 (29%)	149 (26.9%)	216 (24.3%)	
A	789 (45.8%)	535 (48.4%)	886 (49.9%)	0.053
G	933 (54.2%)	571 (51.6%)	890 (50.1%)	

PTC: papillary thyroid cancer; NG: nodular goiter; NC: normal control.

**Table 2:** Association between *MET* SNPs (rs1621 and rs6566) and thyroid tumors as stratified by the gender.

*MET*	Male				Female			
	PTC (*n* = 208)	NG (*n* = 137)	NC (*n* = 220)	*P* value	PTC (*n* = 649)	NG (*n* = 418)	NC (*n* = 676)	*p* value
rs1621								
AA	165 (79.3%)	113 (82.5%)	175 (79.5%)	0.902	491 (75.7%)	335 (80.1%)	549 (81.2%)	**0.037**
AG	41 (19.7%)	22 (16.1%)	42 (19.1%)		152 (23.4%)^*∗*^	76 (18.2%)	116 (17.2%)	
GG	2 (1%)	2 (1.5%)	3 (1.4%)		6 (0.9%)	7 (1.7%)	11 (1.6%)	
A	371 (89.2%)	248 (90.5%)	392 (89.1%)	0.811	1134 (87.4%)	746 (89.2%)	1214 (89.8%)	0.125
G	45 (10.8%)	26 (9.5%)	48 (10.9%)		164 (12.6%)	90 (10.8%)	138 (10.2%)	
rs6566								
AA	40 (19.1%)	38 (27.5%)	47 (21.7%)	0.364	138 (21.2%)	93 (22.5%)	167 (24.9%)	0.261
AG	112 (53.6%)	65 (47.1%)	119 (54.8%)		320 (49.2%)	207 (50%)	339 (50.5%)	
GG	57 (27.3%)	35 (25.4%)	51 (23.5%)		193 (29.6%)	114 (27.5%)	165 (24.6%)	
A	192 (45.9%)	141 (51.1%)	213 (49.1%)	0.574	596 (45.8%)	393 (47.5%)	673 (50.1%)	0.077
G	226 (54.1%)	135 (48.9%)	221 (50.9%)		706 (54.2%)	435 (52.5%)	669 (49.9%)	

PTC: papillary thyroid cancer; NG: nodular goiter; NC: normal control; ^*∗*^ *P* < 0.01 versus the genotype AG in rs1621 in the NC group.

**Table 3:** Associations between the genotypes of *MET* SNP (rs1621) and risk of PTC in male and female patients.

Gender	Group	OR (95% CI)			OR (95% CI)	
		AA	AG	GG	A	G
Male	PTC	1	1.035 (0.641–1.673)	0.707 (0.117–4.285)	1	0.991 (0.644–1.524)
	NG	1	0.811 (0.46–1.431)	1.032 (0.17–6.276)	1	0.861 (0.525–1.412)

Female	PTC	1	**1.465 (1.118**–**1.92)**	0.61 (0.224–1.661)	1	1.273 (1–1.62)
	NG	1	1.074 (0.78–1.478)	1.043 (0.4–2.716)	1	1.058 (0.805–1.389)

PTC: papillary thyroid cancer; NG: nodular goiter; OR: odds ratio; CI: confidence intervals.

**Table 4:** Association between *MET* rs1621 SNP and metastasis or prognosis of the PTC patients as stratified by the gender.

		Male (*n* = 124)				Female (*n* = 374)			
		AA	GG	GA	*p* value	AA	GG	GA	*p* value
Metastasis	No	59	1	17	0.601	202	3	61	0.392
	Yes	40	0	7		78	0	30	

Prognosis	No	37	1	12	0.154	22	0	8	0.750
	Yes	105	0	23		418	6	123	

PTC: papillary thyroid cancer.
